# Duplication of TBK1 Stimulates Autophagy in iPSC-derived Retinal Cells from a Patient with Normal Tension Glaucoma

**DOI:** 10.4172/2157-7633.1000161

**Published:** 2014-01-25

**Authors:** Budd A. Tucker, Frances Solivan-Timpe, Ben R. Roos, Kristin R. Anfinson, Alan L. Robin, Luke A. Wiley, Robert F. Mullins, John H. Fingert

**Affiliations:** 1 Department of Ophthalmology and Visual Sciences, Carver College of Medicine, University of Iowa, Iowa City, IA 52242, USA; 2 Stephen A. Wynn Institute for Vision Research, Carver College of Medicine, University of Iowa, Iowa City, IA 52242, USA; 3 Department of Ophthalmology, Johns Hopkins University, Baltimore, MD 21287, USA; 4 Bloomberg School of Public Health, Johns Hopkins University, Baltimore, MD 21205, USA

**Keywords:** TBK1, Autophagy, Glaucoma, Stem cells, iPSC, Retinal ganglion cells

## Abstract

Duplication of the*TBK1* gene causes normal tension glaucoma (NTG); however the mechanism by which this copy number variation leads to retinal ganglion cell death is poorly understood. The ability to use skin-derived induced pluripotent stem cells (iPSCs) to investigate the function or dysfunction of a mutant gene product in inaccessible tissues such as the retina now provides us with the ability to interrogate disease pathophysiology *in vitro*. iPSCs were generated from dermal fibroblasts obtained from a patient with *TBK1*-associated NTG, via viral transduction of the transcription factors *OCT4*, *SOX2*, *KLF4*, and *c-MYC*. Retinal progenitor cells and subsequent retinal ganglion cell-like neurons were derived using our previously developed stepwise differentiation protocol. Differentiation to retinal ganglion-like cells was demonstrated via rt-PCR targeted against TUJ1, MAP2, THY1, NF200, ATOH7 and BRN3B and immunohistochemistry targeted against NF200 and ATOH7. Western blot analysis demonstrated that both fibroblasts and retinal ganglion cell-like neurons derived from NTG patients with *TBK1* gene duplication have increased levels of LC3-II protein (a key marker of autophagy). Duplication of *TBK1* has been previously shown to increase expression of *TBK1* and here we demonstrate that the same duplication leads to activation of LC3-II. This suggests that *TBK1*-associated glaucoma may be caused by dysregulation (over-activation) of this catabolic pathway.

## Introduction

Glaucoma is a common cause of vision disability and blindness worldwide [1,2]. A common, defining feature of primary glaucomas is a progressive loss of the retinal ganglion cells and their axons, which form the optic nerve. Elevated intraocular pressure is a major risk factor for primary open angle glaucoma (POAG), however, glaucoma can occur at any intraocular pressure. In normal tension glaucoma (NTG), retinal ganglion cells are lost in the absence of elevated intraocular pressure.

The genetic basis of glaucoma is complex, with some cases due primarily to mutations in single genes (Mendelian forms of glaucoma), while others are due to the combined actions of many genes and environmental factors [3]. Mendelian forms of open angle glaucoma have been associated with mutations in myocilin (*MYOC*) [4], optineurin (*OPTN*) [5], WD repeat domain36 (*WDR36*) [6], neurotrophin 4 (*NTF4*) [7], TANK binding kinase 1 (*TBK1*) [8], and ankyrin binding kinase 10 (*ASB10*) [9]. Mutations in *MYOC* are associated with 3–4% of POAG cases that typically have markedly elevated intraocular pressure [10]. Conversely, mutations in *OPTN* and *TBK1* are associated with 1–2% of cases of NTG that do not have elevated intraocular pressure [5,8]. The role of *WDR36*, *NTF4* and *ASB10* in glaucoma pathogenesis is currently controversial [11–16].

The mechanism by which duplication of *TBK1* causes retinal ganglion cell death and NTG is poorly understood. However, recent data suggests that dysregulation of autophagy, a process by which the intracellular accumulation of proteins, organelles, or pathogens may be eliminated [17], might be important in the pathogenesis of NTG. Autophagy is activated in experimental animal models of glaucoma including optic nerve transection and ocular ischemia model systems [18,19]. Moreover, the NTG genes, *TBK1* and *OPTN*, have both been shown to be important regulators of autophagy. For instance, *TBK1* encodes a kinase that phosphorylates OPTN, which then recruits the microtubule-associated protein 1 light chain 3 beta (MAP1LC3B, LC3B) that is instrumental in assembling the autophagosome and initiating autophagy [20]. Duplication of *TBK1* gene in NTG patients has been shown to cause increased transcription of *TBK1* [8], suggesting that this form of glaucoma may be caused by stimulation of autophagy.

Several eye diseases have been successfully modeled in cell culture, by producing induced pluripotent stem cells (iPSCs) from accessible, non-ocular patient tissues then forcing the stem cells to differentiate into the specific ocular cell type affected by the disease. Photoreceptor cells and retinal pigment epithelial cells from patients with retinal degenerations, retinitis pigmentosa [21,22] and Best disease [23], have been produced from iPSCs and used to study disease mechanism. More, recently, Minegishi and coworkers reported that the over-expression of a glaucoma causing-mutation in *OPTN*, Glu50Lys, produces an accumulation of insoluble OPTN protein that can be blocked with chemical inhibition of TBK1 activity in HEK293 cells [24]. This observation was further investigated by using iPSCs and iPSC-derived neural cells from NTG patients with a Glu50Lys *OPTN* mutation. These studies have confirmed the role of *OPTN* and *TBK1* in the pathophysiology of NTG and suggest that mechanisms to eliminate abnormal proteins and other cellular materials, such as autophagy or the unfolded protein response, may be important in the development of glaucoma. Here we report the development and characterization of iPSCs and retinal ganglion cell-like neurons from unaffected controls and NTG patients with *TBK1* gene duplications to investigate the role of autophagy in the pathogenesis of NTG. This represents the first line of iPSC-derived ocular cells that harbor a glaucoma-causing mutation.

## Methods and Materials

### Patient-derived cells

All experiments were conducted with the approval and supervision of the University of Iowa Internal Review Board (Application #200202022) and were consistent with the Treaty of Helsinki. Skin biopsies were collected from patients after informed consent was obtained and were used for the generation of fibroblasts (isolation performed as described previously) [25–27]. Cells were expanded from patients diagnosed with normal tension glaucoma associated with a *TBK1* gene duplication as previously described or from unrelated control subjects that do not have a diagnosis of glaucoma. All subjects were enrolled at the University of Iowa Department of Ophthalmology and Visual Sciences.

### Generation of iPSCs

iPSCs were generated from human skin cells (fibroblasts) via infection with 4 separate non-integrating Sendai viruses, each of which were designed to drive expression of one of four transcription factors: OCT4, SOX2, KLF4, and c-MYC (Invitrogen, A1378001). Fibroblasts plated on six-well tissue culture plates were infected at a multiplicity of infection of 5. At 12–16 hours post-infection, cells were washed and fed with fresh growth medium [minimal essential medium-α, 10% KnockOut Serum Replacement (KSR) (Invitrogen, Carlsbad, CA, http://www.invitrogen.com), 1% primocin (InvivoGen, San Diego, CA, http://www.invivogen.com)]. At 7 days post-infection, cells were passaged onto 6-well Synthemax^™^ cell culture dishes at a density of 300,000 cells/well, and fed every day with reprogramming media (DMEM F-12 media (Gibco), 20% knockout serum replacement (Gibco), 0.0008% beta-mercaptoethanol (Sigma-Aldrich, St. Louis, MO), 1% 100x NEAA (Gibco), 100 ng/ml bFGF (human) (R&D), and 0.2% primocin (Invivogen)). At 3 weeks post-viral transduction, cultures were transitioned to mTeSRmedia, iPSC colonies were picked, passaged and clonally expanded on fresh Synthemax^™^ plates. A minimum of 3 separate clones was selected for subsequent differentiation and analysis experiments. During reprogramming and maintenance of pluripotency, cells were cultured at 5% CO_2_, 5% O_2_, and 37°C.

### iPSC differentiation

Neurons with retinal ganglion cell features were cultured as previously described [28]. Adult-derived iPSCs were cultured in mTeSRmedium to maintain pluripotency. Differentiation was initiated by removing iPSCs from the culture substrate via manual passage using Stem Passage manual passage rollers (Invitrogen) followed by resuspension in embryoid body (EB) medium [DMEM F-12 medium (Gibco) containing 10% knockout serum replacement (Gibco), 2% B27 supplement (Gibco), 1% N2 supplement (Gibco), 1% L-glutamine (Gibco), 1% 100x NEAA (Gibco), 1% penicillin/streptomycin (Gibco), 0.2% Fungizone (Gibco), 1 ng/ml noggin (R&D Systems), 1 ng/ml Dkk-1 (R&D Systems), 1 ng/ml insulin-like growth factor-1 (IGF-1, R&D Systems), and 0.5 ng/ml bFGF (R&D Systems)], and plating at a density of approximately 50 cell clusters per cm^2^ in ultralow adhesion culture plates (Corning). EBs were removed after 5 days in culture (as described above), washed, and plated at a density of 25–30 EBs per cm^2^ in fresh differentiation medium 1 [DMEM F-12 medium (Gibco), 2% B27 supplement (Gibco), 1% N2 supplement [(Gibco), 1% L-glutamine (Gibco), 1%100X NEAA (Gibco), 10 ng/ml noggin (R&D Systems)], 10 ng/ml Dkk-1 (R&D Systems), 10 ng/ml IGF-1 (R&D Systems), and 10 ng/ml bFGF (R&D Systems)] in six-well Synthemax cell culture plates. Cultures were fed with differentiation medium once every other day for 10 days. Cultures were then fed for 6 days with differentiation medium 2 [differentiation medium 1+10 μM of the Notch signaling inhibitor DAPT (Calbiochem, Gibbstown, NJ, http://www.emdbiosciences.com)]. Cultures were next fed for 12 days with differentiation medium 3 [differentiation medium 2+2 ng/ml of acidic fibroblast growth factor (R&D Systems)]. Finally, cultures were allowed to further differentiate for 60 days in differentiation medium 4 (DMEM F-12 medium [Gibco], 2% B27 supplement [Gibco], 1% N2 supplement [Gibco], 1% L-glutamine [Gibco], 1% 100X NEAA [Gibco]).

### Immunopanning with anti-THY1 antibody

THY1/CD90-positive neurons were isolated form differentiated heterogeneous retinal progenitor cell cultures using CD90 magnetic MicroBeads (MiltenyiBiotec Cat# 130-096-253), MS columns and a MACS magnetic separator as per the manufactures specifications. Briefly, differentiated cultures were trypsinized, counted using a Tali Image based cytometer (Invitrogen), centrifuged, and resuspended at a density of 10^7^ cells/80 ul of media. 20 ul of CD90 microbeads were added and the cell suspension was incubated for 30 minutes at 4°C. Cells were subsequently washed, resuspended and loaded into Macs MS columns placed in a MACs magnet. Columns were washed 3 times to remove unbound Thy1-negative cells. Columns were removed from the MACS magnet and cells were flushed into a separate sterile tube using the provided plunger. Thy1-positive cells were plated back into freshly coated culture dishes and used for subsequent analysis.

### Teratoma formation

To validate that generated iPSCs were pluripotent, teratomas were generated by IM injection of 2.5 × 10^6^ undifferentiated iPSCs into immunodefficient (SCID) mice. After 90 days, tumors were excised, fixed, paraffin embedded, and sectioned.

### Histology

Teratomas were fixed in 10% formalin for 24 hours prior to dehydration and mounting in parafin wax (VWR). Samples were sectioned at 6 μm and H&E staining was performed using standard protocols.

### Immunostaining

Cells were fixed in a 4% paraformaldehyde solution and immunostained as described previously [25,28,29]. Briefly, cells were incubated overnight at 4°C with antibodies targeted against NF200 (Abcam, Cambridge, MA) and ATOH7 (Millipore, Billerica, MA) for retinal ganglion cell genesis. Subsequently, Cy2- or Cy3-conjugated secondary antibodies were used (Jackson Immunochem, West Grove, PA) and the samples were analyzed using confocal microscopy. Microscopic analysis was performed such that exposure time, gain, and depth of field remained constant between experimental conditions.

### Immunoblotting

For Western blot analysis iPSC-derived retinal ganglion cells were homogenized in lysis buffer (50 mM Tris-HCl, pH 7.6, 150 mM NaCl, 10 mM CaCl_2_, 1% triton X-100, 0.02% NaN_3,_ (Sigma-Aldrich)) and centrifuged. Supernatants were isolated and protein concentrations determined using a BCA protein assay (Pierce Chemicals, Rockford, IL). Equivalent amounts of protein (20 μg) were subjected to SDS-PAGE (8–10% acrylamide), transferred to PVDF and probed with primary antibodies targeted against LC3B (Cell Signaling Technology, Danvers, MA) and tubulin (Abcam, Cambridge, MA). Blots were visualized with ECL reagents (GE healthcare, Piscataway, NJ) and exposed to X-ray film (Fisher, Pittsburg, PA) or were visualized and quantified using a gel imager (VersaDoc, BioRad, Hercules, CA).

### RNA isolation and rt-PCR

Total RNA was extracted using the RNeasy Mini-kit (Qiagen, Valencia, CA) following the provided instructions. Briefly, cells were lysed, homogenized and ethanol was added to adjust binding conditions. Samples were spun using RNeasy spin columns, washed, and RNA was eluted using RNase-free water. 1 μg of RNA was reverse transcribed into cDNA using the random hexamer (Invitrogen, Carlsbad, CA) priming method and Omni script reverse transcriptase (Qiagen). All PCR reactions were performed in a 40 μL reaction containing 1x PCR buffer, 1.5 mM MgCl_2_, 0.2 mMdNTPs, 100 ng of DNA, 1.0 U of AmpliTaq Gold (Applied Biosystems, Foster City, CA) and 20 pmol of each gene specific primer. All cycling profiles incorporated an initial denaturation temperature of 94C for 10 min followed by 35 amplification cycles with the following conditions, 30 sec at 94C, 30 sec at annealing temperature of each primer and 1 min at 72C with a final extension at 72°C for 10 min. PCR products were separated by electrophoresis on 2% agarose gels (Invitrogen). Gene specific primers (Invitrogen) are given in Table 1. These rt-RCR assays measure overall gene expression and do not differentiate between endogenous or transgene expression.

### Quantitative PCR assay of *TBK1*

**Figure 1:**
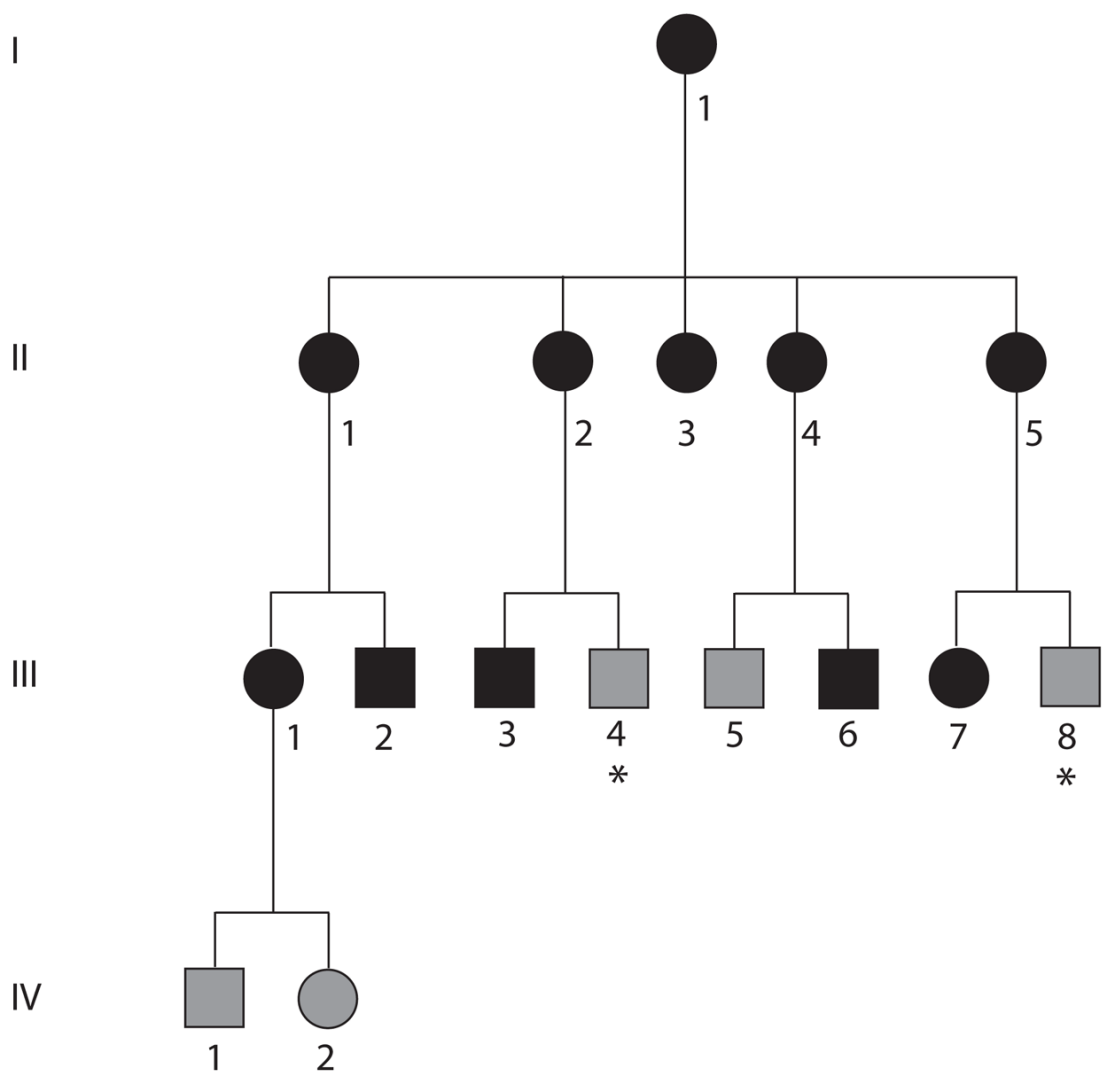
NTG pedigree 441. Members of this African American family with NTG are indicated with black symbols, while those that did not meet diagnostic criteria for glaucoma but were considered to have unknown glaucoma status because of their age are indicated with grey symbols. Family members with unknown glaucoma status because they were unavailable for examination are indicated with grey symbols and asterisks.

*TBK1* copy number was assessed in DNA from white blood cells and iPSC-derived retinal ganglion cells from NTG patient III-1 (Figure 1) known to carry a TBK1 gene duplication and in DNA from the white blood cells of several normal controls using a qPCR assay (TaqMan, BioRad) as previously described [8]. A significant difference was detected between the amount of *TBK1* PCR product produced from the DNA of NTG patients and controls using a t-test (p<0.001).

## Results

### Establishing fibroblast cell lines with TBK1 gene duplication

NTG patients from a large African American pedigree (Pedigree 441, Figure 1) were previously shown to have a 780 kb duplication on chromosome 12q14 that spans *TBK1* using a range of techniques including quantitative PCR (qPCR), SNP analysis, comparative genome hybridization (CGH), and fluorescent *in situ* hybridization (FISH) [8,30]. Fibroblast cells were cultured from skin biopsies obtained from an affected member of pedigree 441 with NTG and the chromosome 12q14 duplication (Figure 1, III-1) using previously described conditions [28]. Fibroblasts were also cultured from two healthy individuals with no *TBK1* duplication as controls.

### Establishing induced pluripotent stem cell (iPSC) lines with *TBK1* gene duplication (iPSC-TBK1)

**Figure 2:**
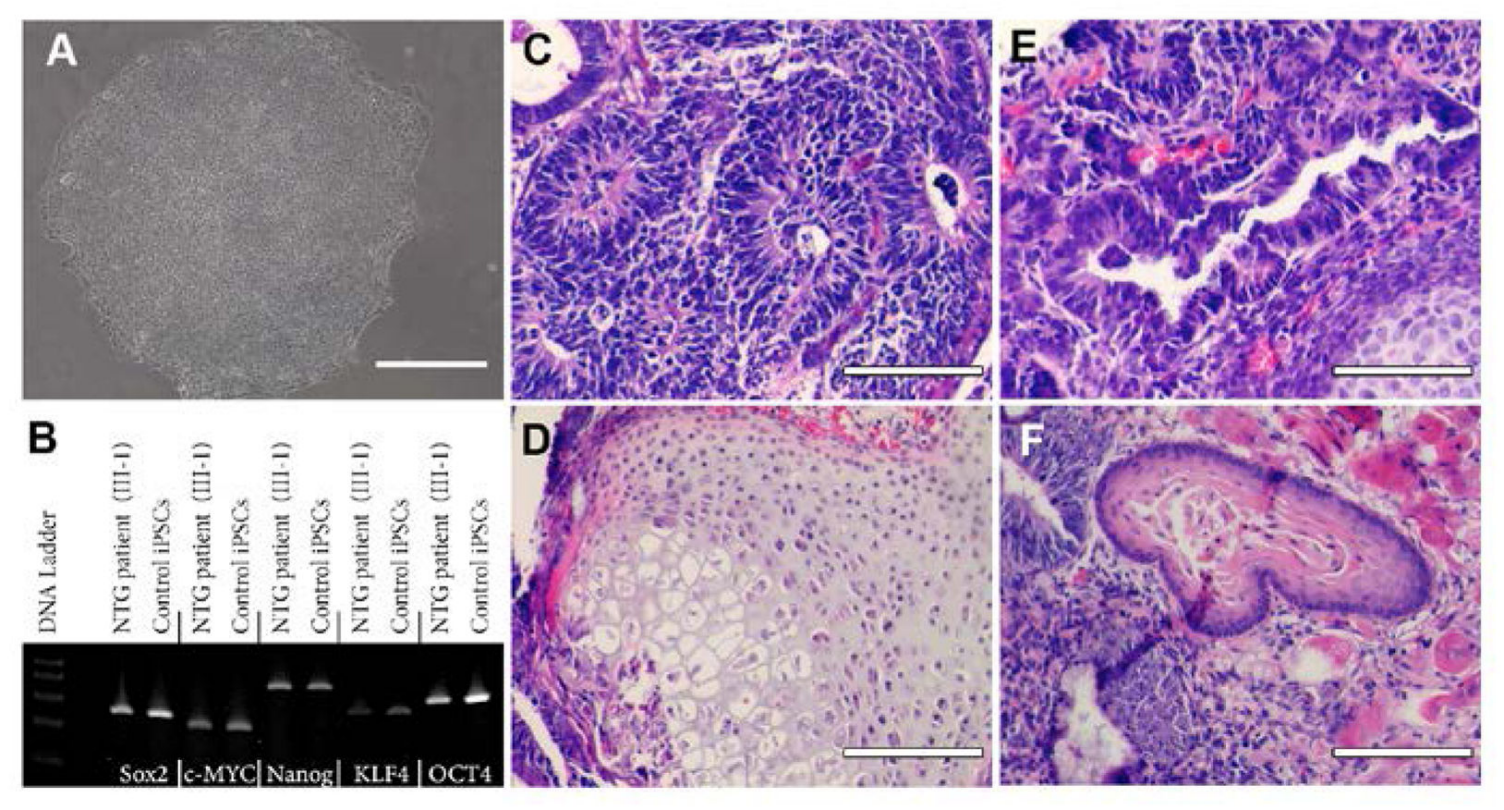
Derivation of iPSCs from a patient affected with *TBK1*-associated Glaucoma. (A) Phase micrograph of a TBK1-iPSC colony demonstrating a pluripotent morphology. (B) rt-PCR analysis of RNA isolated from *WT*-iPSCs and *TBK1*-iPSCs targeted against pluripotency marker expression. (C–F) H&E staining of *TBK1*-iPSC derived teratomas that show each of the three embryonic germ layers ((C) ectoderm, (D) mesoderm, and (E and F) endoderm). Scale bar=100 microns.

Dermal fibroblasts from a family member with NTG and a *TBK1* gene duplication (Figure 1, III-1) were cultured on synthemax cell culture surfaces and reprogrammed via forced expression of the transcription factors *OCT4, SOX2, KLF4*, and *c-MYC*. Two to 3 weeks post-transduction, *iPSCs* colonies containing cells with the typical *iPSC* morphology began to appear. Individual colonies were manually dissected from neighboring fibroblast cells and cultured on Synthemax^™^ [28]. Each colony was expanded for 10 passages at which time cells exhibited morphology typical for stem cells including high nucleus to cytoplasm ratio (Figure 2A). *iPSCs* derived from a *NTG* patient with a *TBK1* gene duplication (Figure 1, III-1) will be referred to as “*TBK1-iPSC*”, while cells derived from healthy volunteers will be referred to as “*WT-iPSC*”. Pluripotency was assessed via *RT-PCR* and teratoma formation assays. Expression of the transcription factors, *SOX2*, *c-MYC*, *NANOG, KLF4*, and *OCT4* was detected (Figure 2B). Moreover, following injection into immune-compromised mice, teratomas containing tissues from all three embryonic germ layers were detected (Figures 2C–2F). Collectively these findings demonstrate the successful generation of pluripotent *iPSCs* from fibroblasts carrying a *TBK1* mutation.

### Differentiation of iPSCs into retinal ganglion-like neurons

**Figure 3:**
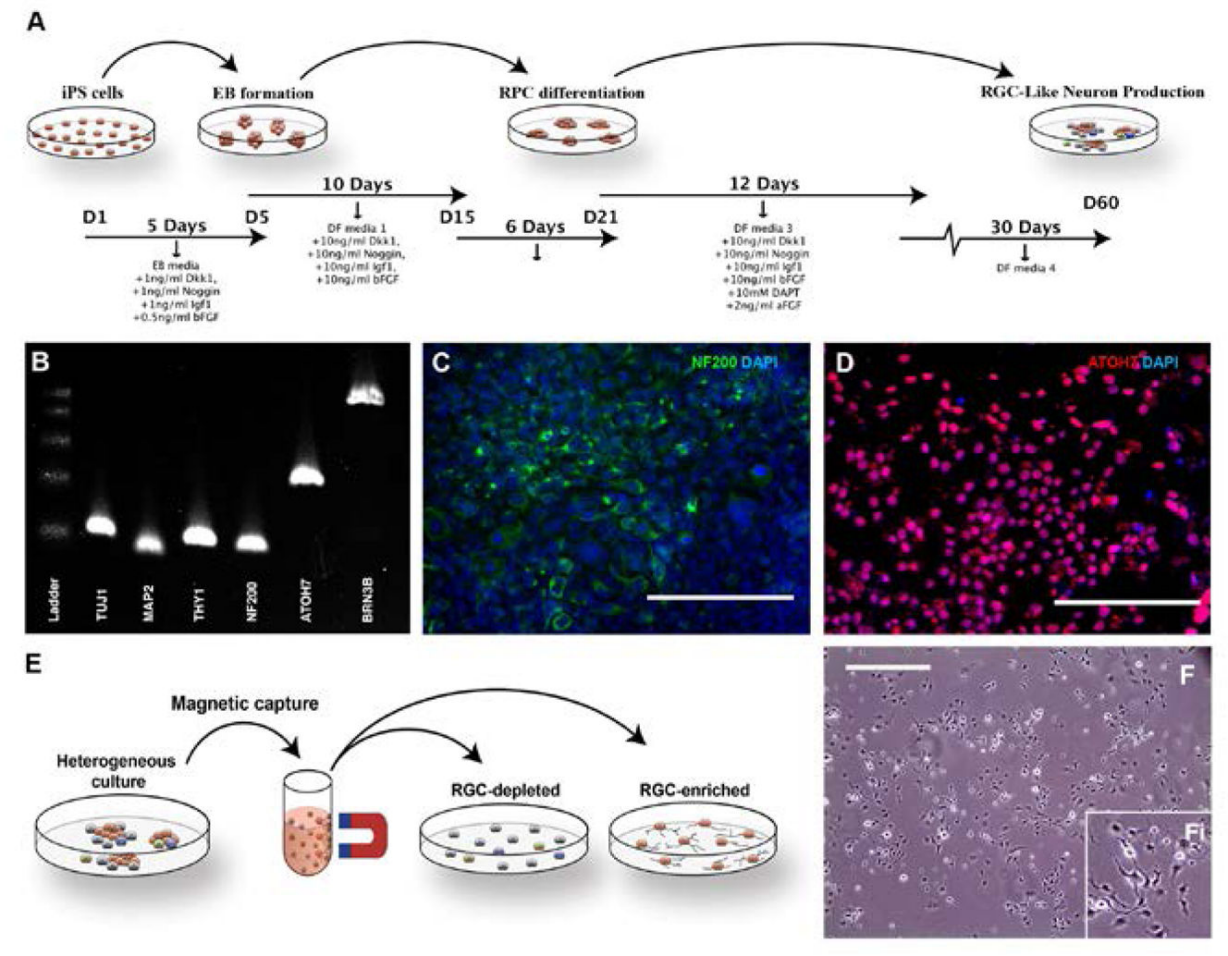
Differentiation of human *TBK1*-associated iPSCs into retinal ganglion cell-like neurons. (A) Schematic of methods used to produce retinal ganglion cell-like neurons. (B) rt-PCR analysis of RNA isolated from *TBK1*-iPSC or *WT*-iPSC derived retinal ganglion cell-like neurons shows expression of markers expressed by RGCs. (C and D) Immunocytochemical analysis of *TBK1*-iPSC derived retinal ganglion cell-like neurons targeted against the neural/retinal ganglion cell markers NF200 (C) and ATOH7 (D). (E) Schematic diagram illustrating the paradigm utilized to isolate/purify TBK1-iPSC derived retinal ganglion cell like neurons from a heterogeneous culture of differentiated cells. (F) Microscopic/morphological analysis of *TBK1*-associated iPSC-derived retinal ganglion cell-like neurons post-magnetic bead isolation. Scale bar=200 microns for panel C and D, and 400 microns for panel F.

**Figure 4:**
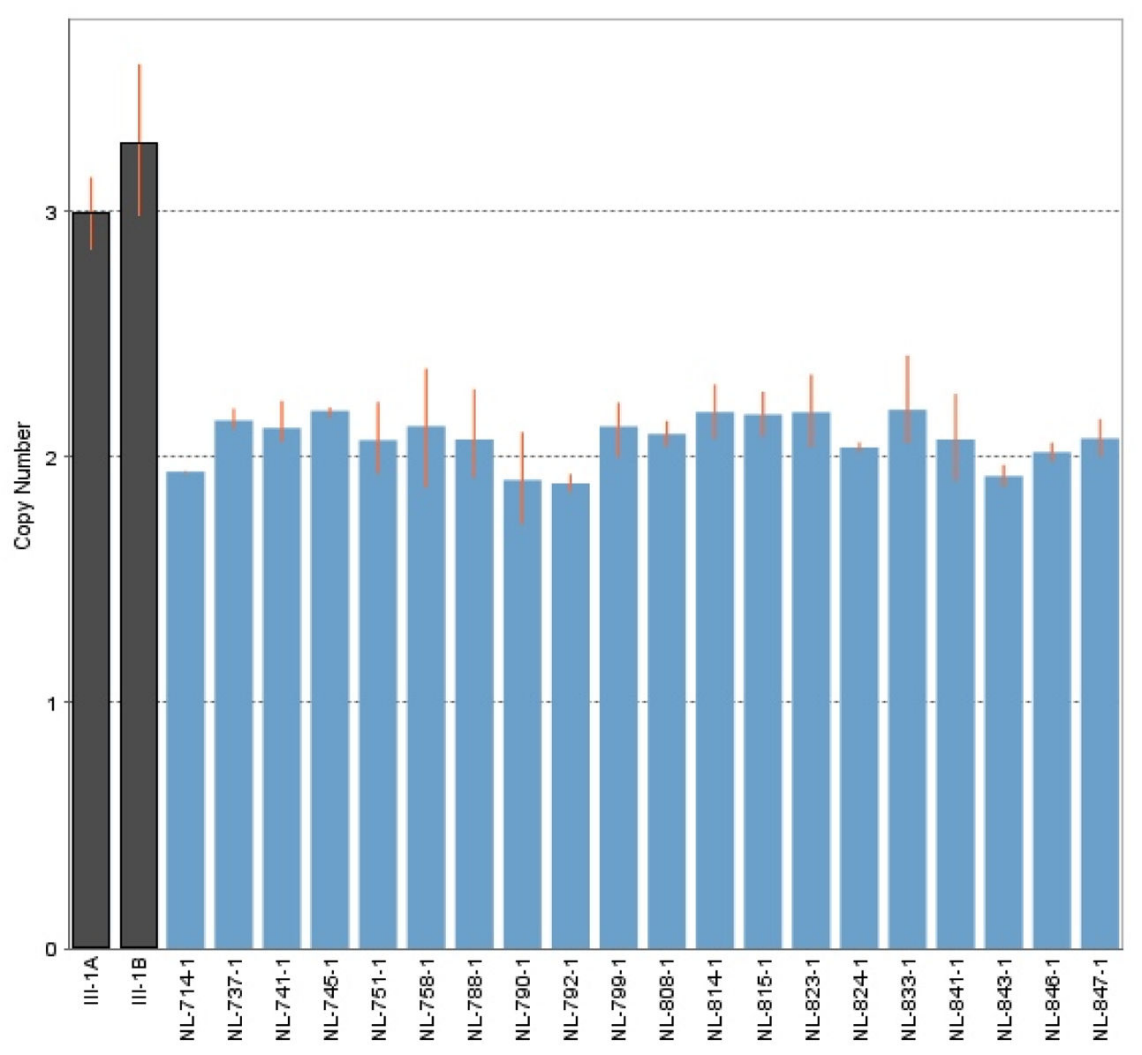
Quantitative PCR assessment of *TBK1* gene dose. The number of copies of the *TBK1* gene was assessed in genomic DNA collected from white blood cells (A) and from iPSC-derived retinal ganglion cells (B) from an NTG patient from family 441 (Figure 1, III-1) and from control subject lymphocytes. This experiment confirms that the iPSC-derived cells carry the*TBK1*-gene duplication originally detected in lymphocytes.

Each of the iPSC lines (TBK1-iPSC and previously generated WT-iPSC lines) were cultured in embryoid body formation media followed by a series of retinal progenitor cell differentiation media containing factors NOGGIN, IGF1, bFGF, DKK-1, and DAPT as outlined in Figure 3A. After 5 days in embryoid body media and a total of 60 days in 4 subsequent different retinal differentiation medias, foci of cells that exhibited morphological features of retinal ganglion cells were observed. These cells were assessed for production of markers that confirm differentiation into retinal ganglion-like neurons. Cells were first tested via *RT-PCR* for expression of the retinal ganglion/neuronal cell markers *TUJ1*, *MAP2, THY1, NF200, ATOH7*, and *BRN3B* (Figure 3B). Subsequently, immunocytochemical staining targeted against *NF200* (Figure 3C) and *ATOH7* (Figure 3D) demonstrated that clusters of retinal ganglion cell like neurons were present post-differentiation. Although retinal ganglion cell like neurons could readily be detected in differentiated cultures, for the purpose of subsequent analysis, *THY1* magnetic bead purifications/*RGC* enrichments were performed as depicted in Figure 3E. Following *THY1* affinity purification, cells were liberated, re-plated in fresh differentiation media, and cultured for an additional 72 hours to allow the cells to recover and redevelop a neuronal morphology (Figure 3F). Finally, we confirmed that cells differentiated from an *NTG* patient (Figure 1, III-1) carry the *TBK1* gene duplication previously detected in *DNA* collected from lymphocytes using quantitative *PCR* (Figure 4). These data indicate that *iPSC*-derived cells from an *NTG* patient carrying a *TBK1* gene duplication, have key features of retinal ganglion cells and may be a useful tool for studying retinal ganglion cell biology and the mechanism of disease in *TBK1*-related glaucoma.

### Assessing activation of autophagy in *iPSC* derived retinal ganglion cells

Our prior studies of NTG suggest that duplication of *TBK1* and increased *TBK1* transcription may cause NTG via overactivation of autophagy [8,20,30]. Consequently we investigated this hypothesis, by testing for activation of autophagy in iPSC-derived retinal ganglion cells that were produced from an NTG patient with *TBK1* gene duplication.

**Figure 5:**
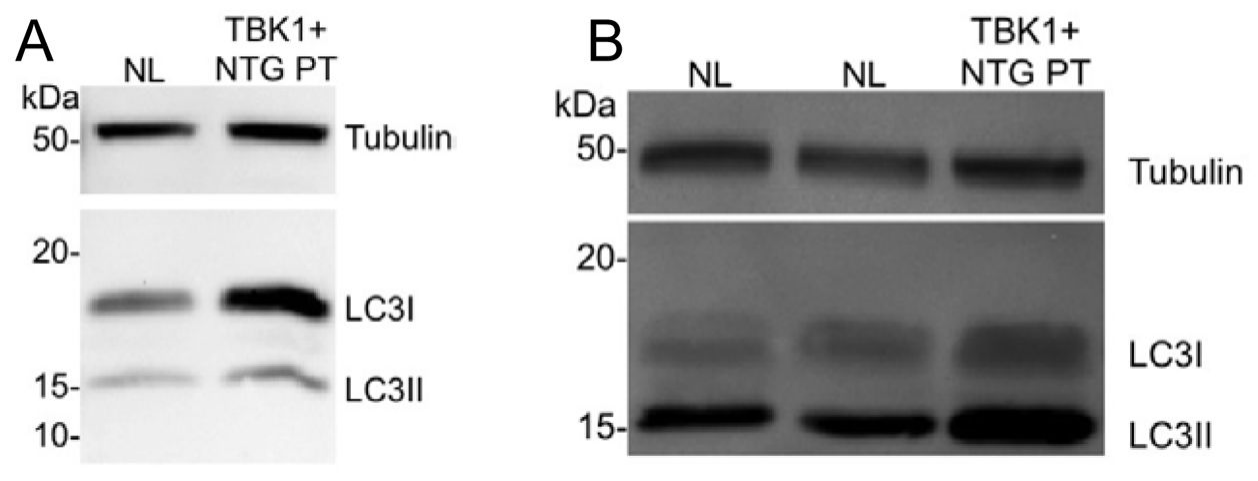
Western blot analysis of fibroblast cells (A) and iPSC-derived retinal ganglion cells (B) with LC3B antibody. LC3-II, the lipidated isoform of LC3, is more abundant in both cell types that carry a *TBK1* gene duplication than in cells with no duplication (NL), but is especially increased in iPSC-derived retinal ganglion cell-like neurons (B). Increased LC3-II suggests that autophagy is activated in iPSC-derived neurons that carry a *TBK1* gene duplication.

Differentiated cells were first purified using THY1 antibody conjugated to magnetic beads. After 72 hrs post-plating cell lysates were collected and examined for the key marker of activation of autophagy, the lipidated form of LC3 (LC3-II), using Western blot analysis. A low level of LC3-II was detected in iPSC-derived retinal ganglion cells from control subjects (Figure 5). However, when compared to a control protein (tubulin), there was a 3-fold increase in LC3-II expression in iPSC-derived retinal ganglion cells from an NTG patient with a *TBK1* gene duplication. These data demonstrate that an extra copy of the *TBK1* gene leads to activation of a critical autophagy protein in the cell type most affected by NTG.

## Discussion

Methods to detect genes that are important in glaucoma pathophysiology have become increasingly successful. Linkage analysis of large pedigrees with Mendelian forms of glaucoma has identified three genes that cause glaucoma (*MYOC*, *OPTN*, and *TBK1*) [4,5,8]. Similarly, genome-wide association studies of large cohorts of patients and controls have detected many risk factors for complex genetic forms of the disease (*CAV1*/*CAV2*, *CDKN2B*-*AS1*, *TMCO1*, *SIX1*/*SIX6*, and others) [3,31–33]. Studying the biological mechanism by which glaucoma genes and risk factors lead to disease, however, has remained a significant challenge. Studies of ocular tissue from patients with glaucoma caused by a particular gene or risk factor are rarely, if ever, possible given the inaccessibility of the retina and optic nerve from living patients and the absence of donor eyes from patients with known genetic causes of their eye disease.

A successful alternative approach has been to study the ocular tissue of transgenic animals that have been engineered to carry the same disease-causing mutations as human patients with glaucoma. For example, studies of transgenic mice have provided key insights into the pathogenesis of glaucoma caused by *MYOC* mutations. Mice engineered to carry a human *MYOC* gene with the Tyr43His mutation develop elevated intraocular pressure and glaucoma that may be related to accumulation of misfolded mutant MYOC protein in key structures of the eye [34,35]. Similar studies of *OPTN*-related glaucoma with transgenic mice are also underway [24,36]. The obvious successes of studying disease mechanism using transgenic mice are also associated with disadvantages, including the high cost of animal experiments that often have lengthy timeframes. Additionally, the genetic, biochemical, and anatomical differences between animal and human eyes may provide other challenges to studies of glaucoma using transgenic animals. While primary cultures of retinal ganglion cells may be isolated from human donor eyes, such research may be limited by the number and purity of cells that may be collected. Consequently, we have developed an iPSC-based approach to obtain relevant cell cultures from living patients that would otherwise be unavailable to study the pathogenesis of glaucoma.

We have modified our previously reported method for differentiating iPSCs into retinal precursors and photoreceptors [22,25,28] to identify and isolate retinal ganglion cell-like neurons using a novel affinity purification method. We first produced and expanded a heterogeneous mixture of retinal cell precursors and differentiated cells, some of which have features of retinal ganglion cells. Those cells that express the retinal ganglion cell surface marker THY1 were then isolated from the heterogeneous cell culture by immuno-panning with anti-THY1 coated magnetic beads. The result is a highly purified homogeneous culture of cells that exhibit morphological features of ganglion cells and express many key markers for neural and retinal ganglion cells including TUJ1, MAP2, NF200, ATOH7, THY1, and BRN3B.

The two genes known to cause Mendelian forms of NTG, *OPTN* and *TBK1*, have been shown to directly interact with each other as key participants in a signaling pathway that activates autophagy. TBK1 phosphorylates OPTN and glaucoma-causing mutations in OPTN have been shown to alter the interaction between TBK1 and OPTN [37,38]. Duplication of the *TBK1* gene has also been shown to increase expression of *TBK1* [8]. Consequently, we have hypothesized that duplication of the *TBK1* gene causes NTG by abnormal activation of autophagy, which may ultimately lead to apoptosis and retinal ganglion cell death. We have used our iPSC-derived retinal ganglion cell-like cultures to begin to explore the mechanism by which *TBK1* gene duplications influence autophagy. Here we show for the first time that mutations in glaucoma-causing genes cause increased expression of LC3-II the key marker of activation of autophagy. These data suggest that over-activation of autophagy may be an important factor in the development of NTG, however, more studies to confirm and extend this hypothesis will be needed to establish the role of autophagy in glaucoma. This cell culture system, however, will provide a powerful means for further dissecting the biochemistry of autophagy in retinal ganglion cells and how dysregulation may lead to retinal ganglion cell death and glaucoma. For example, we may use this cell culture system to explore the effects of TBK1 inhibitors on LC3-II production to further support our autophagy hypothesis. This cell culture resource will also facilitate techniques such as Western blot analysis and other protein-based experiments that would not be possible with cells collected from human donor eyes or with eyes from genetically engineered animals. Large, homogeneous cultures of iPSC-derived retinal ganglion cell-like neurons that carry known glaucoma-causing mutations that are expressed at physiological levels by native promoters are now available for such studies to investigate the pathophysiology of glaucoma.

Minegishi and coworkers recently reported producing iPSCs and iPSC-derived neural cells from tissue samples of an NTG patient with a Glu50Lys *OPTN* mutation [24]. This *OPTN* mutation caused accumulation of abnormal OPTN protein that likely has a role in the development of *OPTN*/TBK1-related NTG and supports our hypothesis that dysregulation of cellular mechanisms that respond to eliminate these proteins (such as autophagy or unfolded protein response) may be an important step in the pathophysiology of this disease.

Current therapies for glaucoma (medications, laser procedures, filtering surgeries, and tube shunts) are all designed to slow or halt disease progression by lowering intraocular pressure. Production of iPSC-derived retinal ganglion cell-like neurons will facilitate development of new classes of glaucoma therapies. This cell culture system will allow large scale testing of pharmacological agents that may identify new compounds that have the ability to alter autophagy. These compounds may have neuroprotective features and prevent the activation of autophagy and subsequent loss of retinal ganglion cells. Such drugs may add to the current armamentarium of glaucoma medications to prevent or slow retinal ganglion cell loss from glaucoma. Retinal ganglion cell-like neurons are also the key reagent needed to develop regenerative, cell-based therapies for end-stage glaucoma.

The insights into glaucoma pathogenesis gained from iPSC-based studies will also help researchers design animal models and test new ideas for drug therapy (i.e. inhibition of autophagy) for their ability to slow or prevent vision loss from progression of glaucoma. Also, retinal ganglion cell-like neurons produced from patients’ own skin may eventually be used to replace neurons in their retina and restore vision that was previously lost to glaucoma. Future studies of these iPSC-derived retinal ganglion cells will be one of many steps towards better treatment options for vision loss caused by glaucoma.

## Figures and Tables

**Table 1:** Gene specific primer sequences used for rt-PCR.

**SOX2**	CAT CAC CCA CAG CAA ATG AC	**TUJ1**	GAT CAG CGT CTA CTA CAA CGA G
	
	GCA AAC TTC CTG CAA AGC TC		GGC CTG AAG AGA TGT CCA AA
**c-MYC**	GCT GCT TAG ACG CTG GAT TT	**MAP2**	AGT CCT GAA AGG TGA ACA AGA GA
	AGC AGC TCG AAT TTC TTC CA		GTG GAG AAG GAG GCA GAT TAG
**Nanog**	TTC TTC CAC CAG TCC CAA AG	**THY1**	GCT CTC CTG CTA ACA GTC TTG
	TTG CTC CAC ATT GGA AGG TT		GAT GGG TGA ACT GCT GGT ATT
**KLF4**	AGA AGG ATC TCG GCC AAT TT	**NF200**	CGT CAT CAG GCC GAC ATT
	AAG TCG CTT CAT GTG GGA GA		ATT GAG CAG GTC CTG GTA TTC
**OCT4**	AAC TCG AGC AAT TTG CCA AGC TCC	**ATOH7**	TCG CAT CAT CAG ACC TAT GG
	TTC GGG CAC TGC AGG AAC AAA TTC		CCG AAC AGG ACA AAC TCA CA

## Abbreviations

ABS10: Ankyrin Binding Kinase 10
ATOH7: Atonal Homolog 7
CAV1: Caveolin 1
CAV2: Caveolin 2
CDKN2B-AS1: Cyclin-dependent Kinase Inhibitor 2B Antisense 1
CGH: Comparative Genome Hybridization
c-MYC: Avian Myelocytomatosis Viral Oncogene Homolog
CNV: Copy Number Variation
iPSC: Induced Pluripotential Stem Cell
Klf4: Kruppel-like Factor 4
LC3: Microtubule-associated Protein 1 Light Chain 3
MAP2: Microtubule-associated Protein 2
MYOC: Myocilin
NF200: Neurofilament, Heavy polypeptide
NTF4: Neurotrophin 4
NTG: Normal Tension Glaucoma
Oct4: POU Domain, Class 5, Transcription factor 1
OPTN: Optineurin
POAG: Primary Open Angle Glaucoma
SNP: Single Nucleotide Polymorphism
SIX1: SIX Homeobox 1
SIX6: SIX Homeobox 6
Sox2: SRY-box 2
TBK1: TANK Binding Kinase 1
THY1: Thymus Cell Antigen 1
TMCO1: Transmembrane and Coiled-coil Domains 1
TUJ1: Neuron-Specific Class III Beta-tubulin
WDR36: WD Repeat Domain36
